# Editorial: The obesity epidemic: Causes, context, prevention

**DOI:** 10.3389/fpubh.2022.1030180

**Published:** 2022-09-26

**Authors:** Peter Congdon, Dickson Amugsi

**Affiliations:** ^1^School of Geography, Queen Mary University of London, London, United Kingdom; ^2^African Population and Health Research Center, Nairobi, Kenya

**Keywords:** obesity, obesogenic, unhealthy food, declining physical activity, nutrition transition

## Introduction

Obesity (body mass index ≥ 30 kg/m^2^) is a growing public health crisis across the world. The available data suggest that the global burden of obesity has more than tripled since 1975 (1). It is particularly high in some first world countries. However, recent data show that obesity is spreading very fast in low-and middle-income countries (LIMICs), and has reached world record level in some of them (2). At the country level, around 42% of adults in the US are obese, compared to estimates four decades earlier of about 13%. Similarly, in the United Kingdom, prevalence has increased to about 25%. In the LMICs, high obesity is apparent in Brazil, China, Egypt, South Africa, India, Indonesia, Mexico, Pakistan and Russia, while prevalence is more than 75% in countries such as Tonga, Samoa, and Kiribati (3). Obesity forecasts (4) are for continuing growth, with the study (Nianogo and Arah) proposing a micro-simulation model for forecasting cohort obesity.

In racial and ethnic minority sub-groups, such as non-Hispanic blacks in the US, obesity has reached half the adult population. There is also considerable variation between socio-economic groups and geographic areas in the obesity burden: for example, the prevalence of adult obesity varies widely across upper tier local authorities in England, ranging from 11 to 40%.

Obesity is a major cause of premature death, is implicated in recent slowing in improved life expectancy (5), and increases the risk of a range of chronic diseases. There is a seven times greater risk of diabetes in the obese as against those of healthy weight, with a three-fold increase in risk for overweight people (6). Elevated body mass index (BMI) is thought to account for 20% of hypertension and coronary-heart disease (6).

There are many contributing factors to obesity in individual behavior patterns, such as poor food choices and over-eating, sedentary lifestyles, and genetic disposition (with heritability estimates of 40–70%). Adverse trends are apparent in both food consumption patterns and activity levels (7). However, the responsible factors extend far beyond individual behavior (8).

Adverse trends show not only in food consumption and activity levels in higher income countries, but in the geographic diffusion of obesity and overweight. The “nutrition transition” characterizes changing food consumption in low and middle income countries, with many facing a “double burden” of obesity and undernutrition, as well as an upturn in non-communicable disease linked to obesity and overweight (9). For example, the special issue paper by Reddy et al. highlights the nutrition transition and its impacts in South Africa.

## Causes: Proximal influences

Excess consumption of less healthy foods and inadequate activity (surplus of energy intake compared to energy expenditure) can be seen as the proximate cause of obesity (8, 10). There is considerable debate around the dietary patterns implicated, whether overall calories intake (the energy balance model) or processed carbohydrates (11, 12).

Distinct dietary pattern subtypes have been identified, such as Western vs. prudent (13). Kopp (14) characterizes the Western diet as containing “large amounts of high-glycemic/high-insulinemic carbohydrate foodstuff like refined cereals, corn, potatoes and sugars (in particular sucrose and fructose), dairy products, as well as high amounts of fat and substantial amounts of protein”. In LMICs, urbanization typically is associated with adoption of Western diet, and associated declines in cardiometabolic health (15).

The Global Burden of Disease study tracking trends in food consumption between 1990 and 2017 in 195 countries estimates that one in five deaths globally are associated with poor diet, with diet contributing to obesity and a range of chronic diseases.

Reduced activity levels are the other main immediate driving factor for increased obesity. In line with many studies looking beyond individual behaviors, the review (16) argues that “a systems approach that focuses on populations and the complex interactions among the correlates of physical inactivity, rather than solely a behavioral science approach focusing on individuals, is the way forward to increase physical activity worldwide”.

## Contextual influences on obesity

The broader distal context to increased obesity is set by policy, and structural “obesogens” of the built, food and social environments. The broader obesogenic context is multifactorial, with the relevance of particular features varying between countries and between subpopulations (17).

Obesity is associated with the emergence of a food industry producing, and marketing (e.g., through advertising), convenient, highly-processed foods. Food advertising content has been linked to growing child obesity (18), and may provide a misleading perspective on nutritional value (19). Food outlets are also increasingly diverse, providing convenience or fast food, with less need for preparing meals at home (20, 21).

A system perspective emphasizes the role of the capitalism in shaping dietary behavior and consumption. Thus, Wells (22) argues that the key to understanding obesity is an “obesogenic niche” caused by the logic of capitalism. Thus, “historically, capitalism contributed to the under-nutrition of many populations through demand for cheap labor. As the limiting factor for economic growth switched to consumption, capitalism has increasingly driven consumer behavior inducing widespread over-nutrition”.

The global food system interacts with local environmental characteristics to create wide variation in obesity levels between populations, and in the obesogenic context (17). For example, obesity, especially through reduced activity, has been linked to urban sprawl. This type of residential dispersal to low density suburban settings—especially in North America and Australasia—is linked to reduced walkability, disconnected street networks, and greater reliance on car use (23, 24).

Influence on activity levels is also the relevant mechanism for studies into green space access and obesity (25), and research into obesity and access to exercise opportunities (26). The Australian study (Jayasinghe et al.) exemplifies research into access to physical activity infrastructure using a seven category breakdown based on the work of Lee et al. (27).

By contrast, access to healthy food and hence dietary influences on obesity are paramount in studies of the food environment (28). Food store type and location is one aspect of the food environment: supermarkets, and fruit and vegetable markets, are associated with improved access to healthy food, as opposed to fast-food restaurants or outlets, small groceries and convenience stores.

## Obesity context and population sub-groups

US studies find population subgroups (income and ethnic groups) differing considerably in access to healthy food outlets (29), while in the UK there are more fast food outlets in deprived areas than in more affluent areas (30). Food deserts have been designated to describe neighborhoods where poverty, poor public transport, and lack of large supermarkets nearby, limit access to affordable fresh fruit and vegetables (31).

Lesser activity levels have also been reported among females than males (32, 33). For example, the Ethiopian study (Biadgilign et al.) investigates gender differences in physical activity among adolescents.

## Obesity prevention

Many interventions to reduce obesity focus on individual behaviors, for example to promote healthy diets (34) or physical activity (35). Most often such interventions involve face-to-face counseling or group therapy combined with recording of dietary intake and physical activity. For example, the study (Yang et al.) considers a longitudinal weight management program in China.

However, advances in technology provide opportunities to deliver eHealth interventions using the web, phone apps and other digital media (36). For example, the systematic review (37) showed that eHealth intervention had potential to effectively promote physical activity in obese adults, while the Research Topic paper (Reddy et al.) mentioned the benefits of eHealth interventions in sub-Saharan contexts as these can reach large populations at low cost.

There is a growing recognition of the importance of population-level interventions and market interventions (38), though the potential for these remains to be fully explored. Thus, the study (39) mentioned that although early child education settings are often an untapped opportunity for supportive nutrition and physical activity changes, while for older children suitable interventions include comprehensive school-based physical activity programs and improved school nutrition environments. Similarly the study (40) estimated that a 20% tax on sugar sweetened drinks would lead to a reduction in the obesity in the UK of 1.3% (around 180,000 people). Studies of the impact of such taxes, where implemented, indicate significant effects on consumption (41).

Often prior consultation with the relevant community will indicate an appropriate design for an intervention. The Saudi study (Almughamisi et al.) is an example of co-identifying actionable priorities for interventions. The importance of the degree of agency in groups targeted by population interventions has also been emphasized (42).

## Author contributions

Text written initially by PC. Subsequent amendments by DA included. Both authors contributed to the article and approved the submitted version.

## Conflict of interest

The authors declare that the research was conducted in the absence of any commercial or financial relationships that could be construed as a potential conflict of interest.

## Publisher's note

All claims expressed in this article are solely those of the authors and do not necessarily represent those of their affiliated organizations, or those of the publisher, the editors and the reviewers. Any product that may be evaluated in this article, or claim that may be made by its manufacturer, is not guaranteed or endorsed by the publisher.
